# Arsenic Trioxide Suppresses Tumor Growth through Antiangiogenesis via Notch Signaling Blockade in Small-Cell Lung Cancer

**DOI:** 10.1155/2019/4647252

**Published:** 2019-04-10

**Authors:** Meng-Hang Yang, Ke-Jie Chang, Bing Li, Wan-Sheng Chen

**Affiliations:** ^1^Department of Respiratory and Critical Care Medicine, Changzheng Hospital, Second Military Medical University, Shanghai 200003, China; ^2^Department of Pharmacy, Changzheng Hospital, Second Military Medical University, Shanghai 200003, China

## Abstract

Small-cell lung cancer (SCLC) is a highly malignant type of lung cancer with no effective second-line chemotherapy drugs. Arsenic trioxide (As_2_O_3_) was reported to exert antiangiogenesis activities against lung cancer and induce poor development of vessel structures, similar to the effect observed following the blockade of Notch signaling. However, there are no direct evidences on the inhibitory effects of As_2_O_3_ on tumor growth and angiogenesis via blockade of Notch signaling in SCLC. Here, we found that As_2_O_3_ significantly inhibited the tumor growth and angiogenesis in SCLC and reduced the microvessel density. As_2_O_3_ disturbed the morphological development of tumor vessels and downregulated the protein levels of delta-like canonical Notch ligand 4 (Dll4), Notch1, and Hes1* in vivo*. DAPT, a Notch signaling inhibitor, exerted similar effects in SCLC. We found that both As_2_O_3_ treatment and* Notch1* expression knockdown resulted in the interruption of tube formation by human umbilical vein endothelial cells (HUVECs) on Matrigel. As_2_O_3_ had no effects on Dll4 level in HUVECs but significantly inhibited the expression of* Notch1* and its downstream gene* Hes1* regardless of Dll4 overexpression or Notch1 knockdown. These findings suggest that the antitumor activity of As_2_O_3_ in SCLC was mediated via its antiangiogenic effect through the blockade of Notch signaling, probably owing to Notch1 targeting.

## 1. Introduction

Small-cell lung cancer (SCLC) is a type of neuroendocrine tumor characterized with rapid growth, aggressive invasion, and early metastasis. It accounts for 10% to 15% of all lung cancers [1]. Although first-line chemotherapy is effective in 80% of patients with SCLC, disease progression is common and no standard drug therapy recommended by the guidelines for second-line chemotherapy is currently available. Thus, there is a pressing need for new therapies for SCLC. Antiangiogenesis is one of the promising strategies [2–4]. Several antiangiogenic agents targeting vascular endothelial growth factor (VEGF) or its receptor (VEGFR), such as bevacizumab, sorafenib, and sunitinib, have been designed and used for cancer treatment [5, 6]. However, the clinical effects of these angiogenesis inhibitors seem unsatisfactory for the treatment of SCLC.

The Notch pathway has been recognized as an important regulator of angiogenesis aside from the VEGF pathway [7]. Evidence suggests the association of the Notch pathway with the occurrence and development of tumors, especially in the regulation of tumor angiogenesis [8–10]. In the Notch pathway, delta-like canonical Notch ligand 4 (Dll4) and the receptor Notch1 are both located on cell membranes, while the signal is transduced via cell-cell interactions. The activation of the Notch pathway results in the upregulation of several target genes, including* Hes1 *[11]. During the process of angiogenesis, the Notch pathway restricts the excessive proliferation of endothelial cells and promotes their participation to form tube structures with an improved function of blood supply. This unique regulatory effect in angiogenesis makes the Notch pathway a promising target for tumor treatment. Dll4 and Notch1 were found to be overexpressed in many types of tumors, including lung cancer [12]. The blockade of the Notch pathway was shown to lead to the poor development of neovascular networks and formation of nonfunctional blood vessels with insufficient perfusion, consequently leading to the inhibition of tumor growth [13].

Arsenic trioxide (As_2_O_3_) is an old drug used in traditional Chinese medicine; its medicinal value was known by people as early as 2,000 years ago. As_2_O_3_ is now used in the treatment of acute promyelocytic leukemia and some solid tumors [14–17]. Our previous research has revealed the suppressive effect of As_2_O_3_ on SCLC growth through the inhibition of tumor angiogenesis. We found that As_2_O_3_ significantly reduced microvessel density (MVD) and induced poor development of vascular structures in NCI-H446 cell xenograft models [18]. We also demonstrated that As_2_O_3_ restricted the tube formation ability of endothelial cells* in vitro* [19]. As the effects of As_2_O_3_ on angiogenesis regulation were similar to those observed with the blockade of the Notch pathway, we suggest that this pathway may be the antiangiogenic target of As_2_O_3_. It has been reported that As_2_O_3_ could downregulate the expression of* Notch1 *and* Hes1* in keratinocytes, glioma cells, and breast cancer [20–23]. In lung cancer, however, no direct evidences of the inhibitory effects of As_2_O_3_ on the Notch pathway have been reported.

In the present study, we established an SCLC xenograft model using NCI-H69 cells to determine the antitumor and antiangiogenic activities of As_2_O_3_ in SCLC. The inhibitory effects of As_2_O_3_ on the Notch pathway were also determined* in vivo*. DAPT that directly blocks Notch signaling by decreasing the activity of *γ*-secretase [24] was used as a positive control. In addition, we revealed the antiangiogenic effects of As_2_O_3_ with an* in vitro* Matrigel assay and demonstrated the possible underlying mechanism using human umbilical vein endothelial cells (HUVECs) transfected with* Dll4* overexpression or* Notch1* knockdown lentivirus. These data would provide further evidences for the antitumor effects of As_2_O_3_ in SCLC.

## 2. Materials and Methods

### 2.1. Cell Culture

The human SCLC cell line NCI-H69 was obtained from the Cell Bank of the Chinese Academy of Sciences (Kunming, Yunnan, China). HUVECs were obtained from the American Type Culture Collection (Manassas, VA, USA). NCI-H69 cells and HUVECs were cultured in Roswell Park Memorial Institute- (RPMI-) 1640 medium (HyClone, Logan City, Utah, USA) and Dulbecco's modified Eagle's medium (DMEM; HyClone, Logan City, Utah, USA), respectively, both supplemented with 10% fetal bovine serum (HyClone, Logan City, Utah, USA) and 1% penicillin-streptomycin (HyClone, Logan City, Utah, USA). Cells were maintained in a humidified atmosphere containing 5% CO_2_ at 37°C [25].

### 2.2. Animal Xenograft Model and Drug Treatment

Male nude mice, aged 6-7 weeks, were purchased from and raised in the Experimental Animal Center of Second Military Medical University (Shanghai, China). NCI-H69 cells suspended in serum-free medium were subcutaneously injected into the right flank of mice (0.2 mL per mouse at a density of 2.5 × 10^7^ cells/mL). After developing tumors 10 days from cell injection, mice were randomly divided into four groups (5 mice per group) and treated with 2.5 or 5.0 mg/kg (i.p.) of As_2_O_3_ (Shuanglu Pharmaceutical, Beijing, China), 10.0 mg/kg of DAPT (Selleck Chemicals, Houston, Texas, USA) (p.o.), or normal saline (i.p.) as control. All agents were administered once every day for 10 days. Tumor volume was calculated as (*a* ×* b*^2^)/2, where* a* and* b* represented the largest and smallest lengths of the tumor, respectively. Tumor growth inhibition (TGI) was calculated with the following equation: TGI = (1 − mean tumor volume of the treated group/mean tumor volume of the control group) × 100%. Animal welfare and experimental procedures were carried out in accordance with the Guide for the Care and Use of Laboratory Animals (Ministry of Science and Technology of China) and the Experimental Animal Ethical Care Guidelines of Second Military Medical University.

### 2.3. Immunohistochemistry and MVD Evaluation

Tissue samples were fixed with 4% paraformaldehyde solution, embedded in paraffin, and sectioned. Sections were deparaffinized, microwaved to optimize antigen retrieval, and blocked with 1% fetal bovine serum and 3% peroxide. Sections were incubated with anti-CD31 primary antibody (1:75, R&D Systems, Minneapolis, Minnesota, USA) overnight at 4°C and a secondary antibody (1:200, KPL, Gaithersburg, Maryland, USA) for 1 h at room temperature. The sections were colored with 3,3′-diaminobenzidine tetrahydrochloride (DAB; DAKO, Carpinteria, California, USA) and counterstained with hematoxylin. The continuous positive CD31 signals represented microvessels in tumor tissues. MVD was determined by counting the number of positive microvessel structures under a microscope in five random fields at 400× magnification.

### 2.4. Western Blot Analysis

The total proteins were extracted from tissues or cells using radioimmunoprecipitation assay (RIPA) lysis buffer, electrophoretically separated, and transferred onto polyvinylidene fluoride (PVDF) membranes. The membranes were blocked and incubated with primary antibodies at 4°C overnight [25]. *β*-actin was used as an internal control. The following primary antibodies were used: Dll4 (1:1000, Abcam, Cambridge, UK), Notch1 (1:1000, Abcam, Cambridge, UK), Hes1 (1:1000, Abcam, Cambridge, UK), and *β*-actin (1:1000, Santa Cruz, Dallas, Texas, USA). After being washed thrice with Tris-buffered saline with Tween, the membranes were incubated with secondary antibodies at room temperature for 1 h and visualized using enhanced chemiluminescence (ECL) detection reagents.

### 2.5. Construction and Transfection of Dll4 Overexpression and Notch1 Knockdown Lentivirus

*Dll4* overexpression gene segment and* Notch1* small-interfering RNA (siRNA) were designed and purchased from GeneChem (Shanghai, China) and cloned into the GV358 and GV248 lentivirus vectors (GeneChem, Shanghai, China), respectively. The appropriate negative control (NC) lentiviruses were also designed. Both lentivirus vectors expressed enhanced green fluorescent protein (GFP) gene. The lentivirus vectors were transfected using Polybrene and Enhanced Infection Solution according to the manufacturer's protocol (GeneChem, Shanghai, China). The transfected cells were confirmed through the evaluation of the expression of GFP under a fluorescence microscope 72 h after transfection. The transfected cells were subsequently expanded to assess* Dll4* upregulation and* Notch1* downregulation.

### 2.6. Real-Time Quantitative Polymerase Chain Reaction (qPCR)

The total RNA was extracted from cells using Trizol reagent (Invitrogen, Carlsbad, California, USA) and reverse-transcribed into cDNA using a ReverTra Ace® Kit (Toyobo, Osaka, Japan) [25]. qPCR was performed using specific primers and Thunderbird RT-PCR Mix (Toyobo, Osaka, Japan). The following primers were used:

Dll4 forward 5′-GTGGGTCAGAACTGGTTATTGGA-3′ and reverse 5′-CTGGCTGGCACACATAGTGG-3′; Notch1 forward 5′-TTTGTGCTTCTGTTCTTCGTGG-3′ and reverse 5′-GAACTTCTTGGTCTCCAGGTCC-3′; *β*-actin forward 5′-GTCCACCGCAAATGCTTCTA-3′ and reverse 5′-TGCTGTCACCTTCACCGTTC-3′. The relative expression level of target mRNA was calculated after normalization with the expression of *β*-actin based on the ΔCt method.

### 2.7. *In Vitro* Vascular Tube Formation Assay

Unpolymerized Matrigel (BD Biosciences, Franklin Lakes, NJ, USA) was placed in 24-well plates (300 *μ*L/well) and allowed to polymerize for 1 h at room temperature. A 500 *μ*L suspension of HUVECs was seeded onto the polymerized Matrigel at a density of 5 × 10^4^ cells/well. HUVECs were treated with 0 or 2.0 *μ*M of As_2_O_3_ in triplicate. After incubation at 37°C for 18 h, images of tube formation were acquired with an inverted phase-contrast microscope. The degree of tube formation was quantified by counting the number of cord structures in five random fields from each well at 40× magnification.

### 2.8. Statistical Analysis

All data were presented as means ± standard deviation (SD). Differences between groups were analyzed with one-way analysis of variance (ANOVA), followed by least significant difference (LSD)* t*-test using SPSS 22.0 software. A value of* P* less than 0.05 was considered statistically significant.

## 3. Results

### 3.1. As_2_O_3_ Suppressed SCLC Xenograft Growth

**Figure 1:**
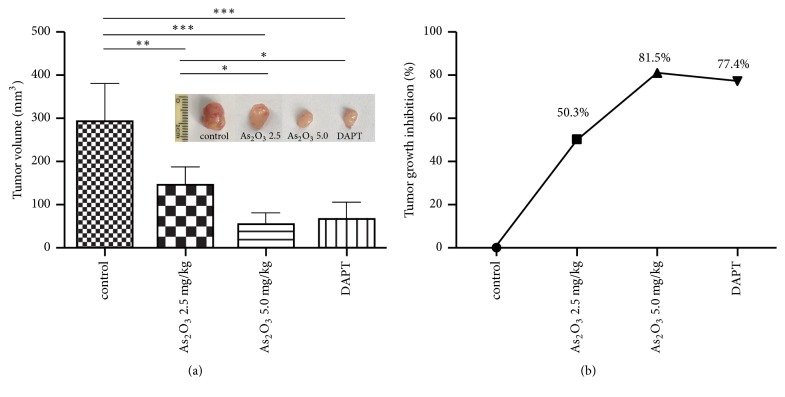
Both As_2_O_3_ and Notch inhibitor suppressed NCI-H69 xenograft growth. (a) Mean tumor volumes of all groups at the end of drug treatment.* Middle panel*, representative images of tumors from each group. (b) Tumor growth inhibition (TGI) reported in all groups at the end of drug treatment.* Columns*, mean;* error bars*, SD. ^*∗*^*P* < 0.05, ^*∗∗*^*P* < 0.01, ^*∗∗∗*^*P* < 0.001.

To determine whether As_2_O_3_ inhibits the growth of SCLC, xenograft tumor models were established using the SCLC cell line NCI-H69. After tumor development, mice were randomly divided into four groups and treated with 2.5 or 5.0 mg/kg of As_2_O_3_, 10.0 mg/kg of DAPT, or normal saline (control) once daily for 10 consecutive days. At the end of treatment, the average tumor volume was significantly smaller in the mice from the two As_2_O_3_ groups than in those from the control group (*P*<0.01 and* P*<0.001, resp.) (Figure 1(a)), and tumor volume was significantly smaller in the mice from the high (5.0 mg/kg) As_2_O_3_ dose group than in those from the low (2.5 mg/kg) As_2_O_3_ dose group (*P*<0.05). The treatment with DAPT, the Notch signaling inhibitor, also resulted in obvious inhibitory effects on tumor growth. The mean tumor volume was smaller in the mice from the DAPT group than in those from the control group (*P*<0.001) and 2.5 mg/kg As_2_O_3_ group (*P*<0.05) but slightly larger than the mice from the 5.0 mg/kg As_2_O_3_ group (Figure 1(a)). As shown in Figure 1(b), the TGI in the 2.5 and 5.0 mg/kg As_2_O_3_ groups and the DAPT group was 50.3%, 81.5%, and 77.4%, respectively. These results suggest that As_2_O_3_ inhibited SCLC growth in a dose-dependent manner, while DAPT treatment also suppressed the growth of SCLC.

### 3.2. As_2_O_3_ Inhibited Tumor Angiogenesis and the Notch Pathway in SCLC Xenografts

**Figure 2:**
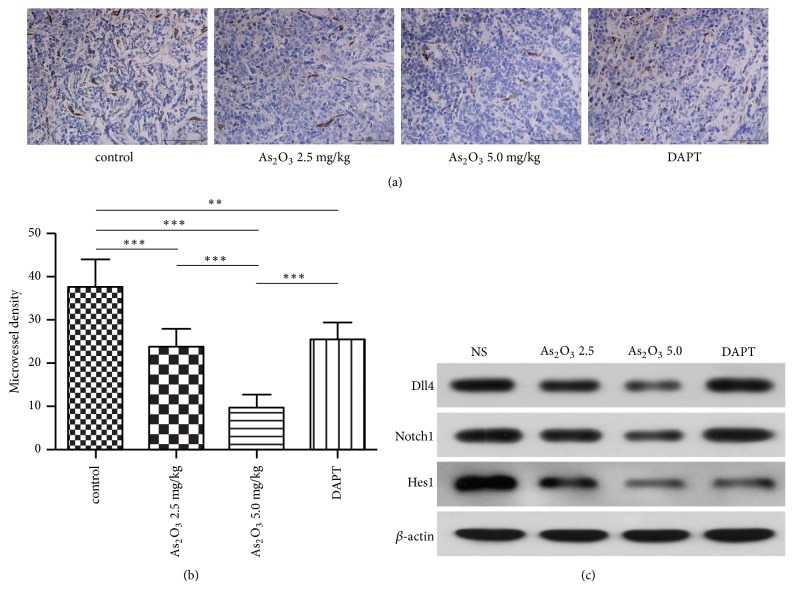
As_2_O_3_ and Notch inhibitor inhibited tumor angiogenesis and the Notch pathway in NCI-H69 xenografts. (a) As_2_O_3_ and DAPT decreased the number of normal microvessel structures. Xenograft sections were immunostained with anti-CD31 antibody, which colored the endothelial cells* brown*.* Scale bars*, 50 *μ*m. (b) Quantification of microvessel density (average of microvessels per field) in each group. (c) Western blot analysis demonstrated the effect of As_2_O_3_ and DAPT treatment on the expression of Dll4, Notch1, and Hes1 at the protein level.* Columns*, mean;* error bars*, SD. ^*∗∗*^*P* < 0.01, ^*∗∗∗*^*P* < 0.001.

To evaluate the effect of As_2_O_3_ treatment on tumor angiogenesis in SCLC, we performed immunohistochemistry for CD31 to determine the number and morphology of microvessels in the xenograft sections from each group. As shown in Figure 2(a), the xenografts from the control group showed high MVD with regular vessel structures. In contrast, the xenografts from As_2_O_3_ treatment groups showed an obvious decrease in MVD with narrow and tortile lumens. The xenografts from the DAPT treatment group showed more single positive signals but decreased normal microvessel structures. The quantification of MVD revealed the inhibitory effect of As_2_O_3_ treatment on tumor angiogenesis in a dose-dependent manner. Although DAPT induced more single positive signals, the MVD in the DAPT group was still lower than that observed in the control group (*P*<0.01) (Figure 2(b)). It was known that the Notch pathway is involved in tumor angiogenesis and may regulate the number and morphological development of vessels. Hence, we examined the expression of the Notch pathway-related factors in xenograft tissues from each group. As shown in Figure 2(c), DAPT treatment downregulated the protein level of Hes1 but had no effect on the expression of Dll4 and Notch1. On the other hand, As_2_O_3_ treatment induced a dose-dependent downregulation in the protein levels of Hes1, Dll4, and Notch1. These data suggest that both As_2_O_3_ and DAPT could inhibit Notch signaling probably through different mechanisms.

### 3.3. Transfection with Specific Lentiviruses Upregulated Dll4 Expression and Downregulated Notch1 Levels in HUVECs

**Figure 3:**
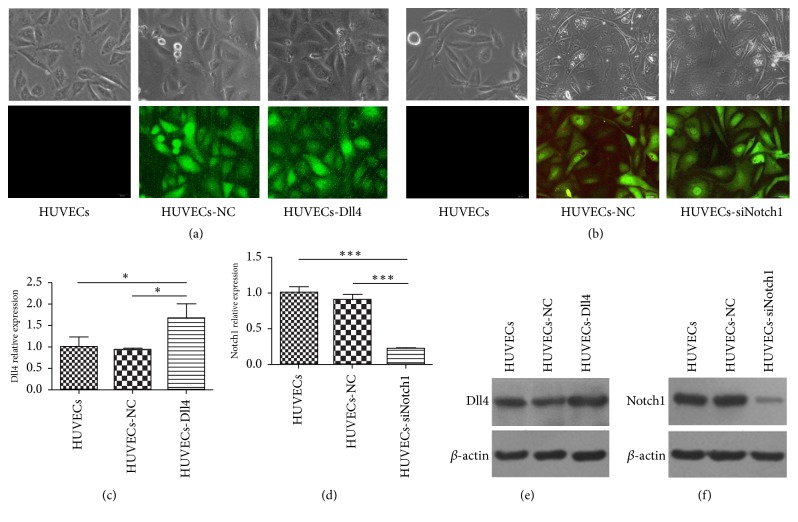
Transfection of* Dll4* overexpression lentiviruses and* Notch1* siRNA lentiviruses into HUVECs. The morphology (*upper panels*) and green fluorescence expression (*lower panels*) of HUVECs with or without transfection of Dll4 overexpression (a) or siNotch1 lentiviruses (b). (c) Transfection of Dll4 overexpression lentiviruses resulted in an increase in* Dll4* mRNA level in HUVECs. (d) Transfection of* Notch1* siRNA lentiviruses reduced the* Notch1* mRNA level in HUVECs. (e) Transfection of* Dll4* overexpression lentiviruses upregulated Dll4 protein level in HUVECs. (f) Transfection of* Notch1* siRNA lentiviruses reduced Notch1 protein level in HUVECs.* Columns*, mean;* error bars*, SD. ^*∗*^*P* < 0.05, ^*∗∗∗*^*P* < 0.001.

To determine the transfection efficiency of the constructed lentiviruses, we used* Dll4* overexpression lentiviruses and* Notch1* siRNA lentiviruses along with their respective negative control (NC) lentiviruses to infect HUVECs. Cell morphology and fluorescence expression were observed under a fluorescence microscope, and the expression of target genes at mRNA and protein levels was measured. As shown in Figures 3(a) and 3(b), the cells transfected with lentiviruses showed green fluorescence under a fluorescence microscope at a transfection efficiency of over 80%. qPCR results showed that the* Dll4* mRNA level was significantly higher in the* Dll4* overexpression lentivirus group than that in the NC lentivirus and blank control groups (*P*<0.05) (Figure 3(c)), while* Notch1* mRNA level was significantly lower in the siNotch1 group than that in the other two groups (*P*<0.001) (Figure 3(d)). Western blot analysis revealed that the Dll4 protein level in the* Dll4* overexpression lentivirus group was significantly higher than that in the other two groups (Figure 3(e)), while the Notch1 protein level in the siNotch1 group was significantly lower than that in the other two groups (Figure 3(f)). These data suggest that the lentiviruses we constructed were efficient in upregulating* Dll4* or downregulating* Notch1* expression in HUVECs.

### 3.4. As_2_O_3_ Disrupted the Tube Formation Ability of HUVECs on Matrigel

**Figure 4:**
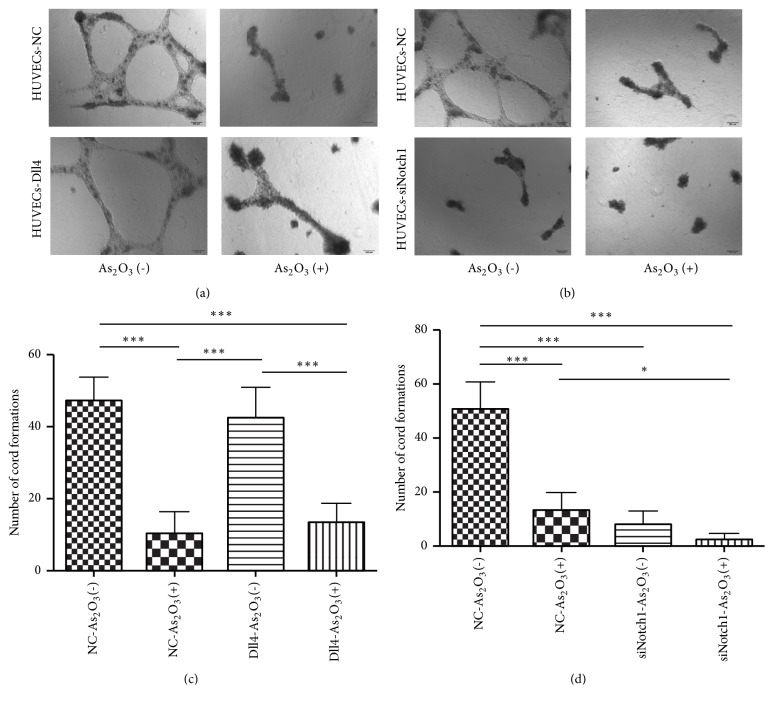
As_2_O_3_ disrupted the tube formation ability of HUVECs* in vitro*. (a) The tube formation capacity of HUVECs transfected with* Dll4* overexpression lentiviruses or NC lentiviruses with or without As_2_O_3_ treatment.* Scale bars*, 200 *μ*m. (b) The tube formation capacity of HUVECs transfected with* Notch1* siRNA lentiviruses or NC lentiviruses with or without As_2_O_3_ treatment.* Scale bars*, 200 *μ*m. (c, d) Quantification of cord formation.* Columns*, mean;* error bars*, SD. ^*∗*^*P* < 0.05, ^*∗∗∗*^*P* < 0.001.

We examined whether As_2_O_3_ could disrupt endothelial tube formation with the Matrigel assay. HUVECs transfected with* Dll4* overexpression lentiviruses,* Notch1 *siRNA lentiviruses, or respective NC lentiviruses were seeded onto Matrigel. The cells were treated with 0 or 2.0 *μ*M As_2_O_3_ for 18 h, and the microphotographs were obtained. As shown in Figures 4(a) and 4(b), HUVECs infected with NC lentivirus could form cross-linked vascular networks in the absence of As_2_O_3_ treatment but failed to form these structures upon As_2_O_3_ treatment. On the other hand, the HUVECs overexpressing* Dll4* could also form vascular networks in the absence of As_2_O_3_ but failed to exhibit this characteristic after As_2_O_3_ treatment (Figure 4(a)). Quantitative analysis showed that As_2_O_3_ significantly decreased the tube formation ability of the HUVECs transfected with NC lentiviruses or* Dll4* overexpression lentiviruses (*P*<0.001) (Figure 4(c)). After* Notch1* knockdown, HUVECs could not form networks even in the absence of As_2_O_3_, and the isolated cord structures disappeared after As_2_O_3_ treatment (Figure 4(b)). Quantitative analysis showed that both* Notch1* knockdown and As_2_O_3_ treatment significantly decreased the tube formation ability of HUVECs (*P*<0.001), and the inhibitory effect was stronger in the presence of the two factors (Figure 4(d)). These results suggest that As_2_O_3_ could inhibit the tube formation ability of vascular endothelial cells, similar to the effect observed with* Notch1* knockdown. The overexpression of* Dll4* could not reverse the inhibition of tube formation by As_2_O_3_.

### 3.5. As_2_O_3_ Inhibited the Expression of Notch1 and Hes1 in HUVECs

**Figure 5:**
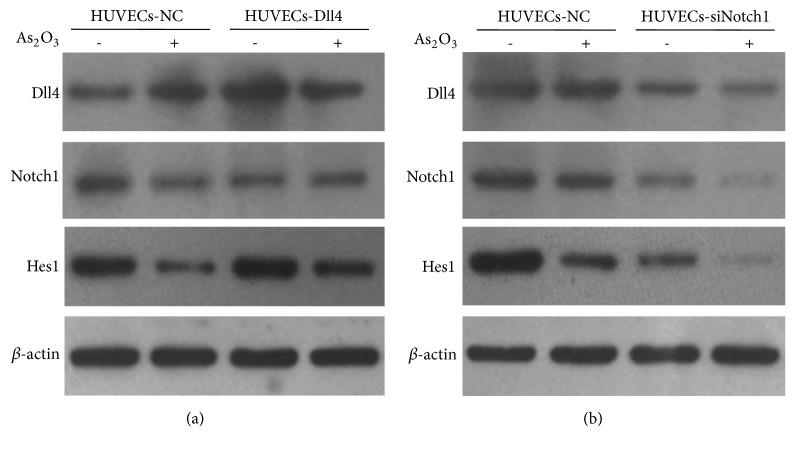
The effect of As_2_O_3_ treatment on the regulation of the expression of the Notch pathway-related factors in HUVECs. (a) Western blot analysis to reveal the expression of Dll4, Notch1, and Hes1 proteins in the HUVECs transfected with Dll4 overexpression lentiviruses or NC lentiviruses with or without As_2_O_3_ treatment. (b) The expression of Dll4, Notch1, and Hes1 proteins in the HUVECs transfected with Notch1 siRNA lentiviruses or NC lentiviruses with or without As_2_O_3_ treatment.

To demonstrate the possible mechanism underlying the inhibitory effects of As_2_O_3_ on angiogenesis, HUVECs were transfected with* Dll4* overexpression lentiviruses,* Notch1* siRNA lentiviruses, or respective NC lentiviruses. The cells were treated with 0 or 2.0 *μ*M of As_2_O_3_ for 48 h, and the expression of key factors involved in the Notch pathway was determined by western blotting. As shown in Figures 5(a) and 5(b), the regulatory effects of As_2_O_3_ on* Dll4* expression were not obvious or caused slight* Dll4* upregulation in HUVECs transfected with NC lentiviruses. However, As_2_O_3_ significantly inhibited the expression of* Notch1* and its downstream target gene* Hes1*. For HUVECs overexpressing* Dll4*, As_2_O_3_ downregulated* Hes1* expression (Figure 5(a)). Notch1 protein expression was downregulated by about 70% in the HUVECs transfected with Notch1 siRNA lentiviruses. As_2_O_3_ treatment enhanced the inhibitory effect of* Notch1* siRNA on* Notch1* and* Hes1* but showed no obvious effect on* Dll4* expression (Figure 5(b)). These results suggest that As_2_O_3_ may block the Notch pathway through the inhibition of Notch1 expression and consequently disturb the process of angiogenesis.

## 4. Discussion

Although As_2_O_3_ is known to exert antitumor activity in some solid tumors both* in vitro* and* in vivo*, it has not yet been widely used in clinical practice possibly owing to the lack of complete information on its functional mechanism of action. Angiogenesis plays a crucial role in the pathophysiological process of malignant diseases and is essential for tumor growth and metastasis. Therefore, antiangiogenesis has been considered as an important therapeutic strategy for the treatment of solid tumors such as lung cancer [26, 27]. In the present study, we established an SCLC xenograft model with NCI-H69 cells and found that As_2_O_3_ treatment could significantly inhibit the tumor growth in a dose-dependent manner. We also demonstrated the antiangiogenic effect of As_2_O_3_ in SCLC tissues. As_2_O_3_ not only reduced MVD but also influenced the morphology of blood vessels by inducing the formation of irregular vascular structures with narrow and tortile lumens. Our previous study showed that As_2_O_3_ inhibited angiogenesis in lung cancer via the downregulation of VEGF signaling [18, 19] and was accountable for the reduction in microvessels. However, we were unable to explain the change in vessel morphology. In the present study, we found that the inhibitor of Notch signaling, DAPT, exhibited antitumor and antiangiogenic activities similar to those of As_2_O_3_* in vivo*. Hence, we speculate that the inhibitory effect of As_2_O_3_ on SCLC may be associated with the blocking of Notch signaling. We determined the protein level of the Notch pathway-related factors in tumor tissues and found that As_2_O_3_ reduced the protein levels of Dll4, Notch1, and Hes1* in vivo*. This result was consistent with our hypothesis.

Notch signaling is a highly conserved pathway in humans and known to regulate a variety of biological functions throughout the embryonic and adult stages [11]. During the classical activation of Notch signaling, the Notch receptors bind to their ligand Dll4 located on neighboring cell membranes and undergo two consecutive hydrolysis steps, resulting in the activation of the Notch intracellular domain (NICD). NICD enters the nucleus, interacts with the related transcription factors and coactivators, and finally activates the downstream genes [28]. In mammals, the most frequently activated downstream genes are* Hes* and* Hey *[29]. The Notch pathway has long been recognized as an indispensable regulator of angiogenesis. Of the four Notch receptors, Notch1 and Notch4 are expressed on endothelial cells [30, 31]. Gene targeting studies in mice have demonstrated Notch1 as the primary functional Notch receptor during developmental angiogenesis [32]. Researchers have constructed animal models with knockdown or overexpression of the Notch pathway-related genes such as* Notch1*,* Dll4*, and* Hes1* to reveal the unique regulatory effects of the Notch pathway on angiogenesis, including normal vascular lumen formation while eliminating the excessive nonfunctional angiogenesis [32–37]. Of note, the Notch pathway was confirmed to be involved in the regulation of tumor angiogenesis. In mouse sarcoma models,* Dll4* knockout or Dll4 blocking antibodies suppressed tumor growth and induced poor development of blood vessels, lumen shutdown, and insufficient blood perfusion [38, 39]. In mouse breast cancer models, Dll4 monoclonal antibody induced the formation of nonfunctional blood vessels in tumor tissues and inhibited the growth of breast cancer [40]. Similar results could be observed following the inhibition of Notch, the receptor of Dll4 [41, 42]. It was also reported that cyclin-dependent kinase 5 (CDK5) was involved in the regulation of the Notch pathway in tumor angiogenesis. The inhibition of CDK5 expression was shown to reduce the formation of NICD, resulting in nonproductive angiogenesis and a decrease in tumor growth [43]. These results suggest that the blockade of Notch signaling may disturb the morphology and functional development of blood vessels in tumor tissues and consequently inhibit tumor growth.

We have previously found that As_2_O_3_ inhibited VEGF signaling in lung cancer [18, 19]. As seen with our* in vivo* study, As_2_O_3_ reduced the protein level of Dll4, Notch1, and Hes1 in SCLC tissues. We investigated whether As_2_O_3_ inhibits the Notch pathway directly or indirectly as a consequence of downregulation of VEGF signaling. We performed additional* in vitro* assays using HUVECs (without extra VEGF secretion in the experimental system) to demonstrate the direct regulatory effect of As_2_O_3_ on endothelial cells and the possible mechanism. To determine the target of As_2_O_3_ in the Notch pathway of endothelial cells, we designed* Dll4* overexpression lentiviruses and* Notch1* siRNA lentiviruses and carried out the* in vitro* tube formation study. Our data showed that both As_2_O_3_ treatment and* Notch1* knockdown disturbed the tube formation ability of HUVECs, while* Dll4* overexpression failed to reverse the disturbing effect of As_2_O_3_. These observations suggest that As_2_O_3_ may prevent the endothelial cells from forming lumen structures through the inhibition of Notch1. To further demonstrate the regulatory mechanism of As_2_O_3_ on Notch signaling, we analyzed the protein levels of Dll4, Notch1, and Hes1 in HUVECs after As_2_O_3_ treatment. As_2_O_3_ treatment had no obvious effect on* Dll4* expression in HUVECs but significantly inhibited the expression of* Notch1* and its downstream gene* Hes1*. In HUVECs overexpressing* Dll4*, As_2_O_3_ could downregulate Hes1 expression, while in the HUVECs transfected with* Notch1* siRNA lentiviruses, Notch1 protein expression was not completely suppressed but was downregulated by about 70%. We observed that As_2_O_3_ enhanced the inhibitory effect of Notch1 knockdown on Notch1 and Hes1. These data suggest that As_2_O_3_ disturbed the tube formation ability of endothelial cells through the inhibition of Notch1 rather than Dll4.

The Notch pathway is recognized as a regulator of angiogenesis downstream of VEGF signaling and provides negative feedback to reduce the overactivation of VEGF signaling [12, 44]. In combination with the results of our previous studies, we found that As_2_O_3_ inhibits VEGF secretion from tumor cells and may subsequently reduce Notch signaling in endothelial cells, although As_2_O_3_ exerted direct effects on Notch signaling in endothelial cells. The interplay between the role of As_2_O_3_ in VEGF and Notch signaling remains to be elucidated in the future.

In conclusion, the present study demonstrates that As_2_O_3_ treatment inhibited tumor growth and angiogenesis and downregulated the Notch pathway in SCLC mouse models. As_2_O_3_ disturbed the tube formation ability of endothelial cells through the inhibition of* Notch1*. Taken together, our data suggest that the antitumor activity of As_2_O_3_ in SCLC was mediated via its antiangiogenic effect through the blockade of Notch signaling, probably by Notch1 targeting. We believe that these findings may provide a foundation for the application of As_2_O_3_ in the treatment of SCLC.

## Data Availability

All data included in this study are available upon request by contacting with the corresponding authors.
